# Nuclear Receptors, Ligands and the Mammalian B Cell

**DOI:** 10.3390/ijms21144997

**Published:** 2020-07-15

**Authors:** Bart G. Jones, Rhiannon R. Penkert, Sherri L. Surman, Robert E. Sealy, Julia L. Hurwitz

**Affiliations:** 1 Department of Infectious Diseases, St. Jude Children’s Research Hospital, Memphis, TN 38105, USA; bart.jones@stjude.org (B.G.J.); rhiannon.penkert@stjude.org (R.R.P.); sherri.surman@stjude.org (S.L.S.); bob.sealy@stjude.org (R.E.S.); 2 Department of Microbiology, Immunology and Biochemistry, University of Tennessee Health Science Center, Memphis, TN 38163, USA

**Keywords:** estrogen, vitamins, immunoglobulin, regulatory elements

## Abstract

Questions concerning the influences of nuclear receptors and their ligands on mammalian B cells are vast in number. Here, we briefly review the effects of nuclear receptor ligands, including estrogen and vitamins, on immunoglobulin production and protection from infectious diseases. We describe nuclear receptor interactions with the B cell genome and the potential mechanisms of gene regulation. Attention to the nuclear receptor/ligand regulation of B cell function may help optimize B cell responses, improve pathogen clearance, and prevent damaging responses toward inert- and self-antigens.

## 1. Nuclear Receptors, Ligands, and the Mammalian Cell

The steroid/thyroid hormone nuclear receptors comprise a superfamily of transcription factors characterized by a DNA-binding domain (DBD), a ligand-binding domain (LBD), and a transactivation domain [1,2]. Type I receptors (including estrogen receptor (ER), glucocorticoid receptor (GR), progesterone receptor (PR), and androgen receptor (AR)) are typically homodimers, while type II receptors are typically heterodimers. Examples of type II receptors include retinoic acid receptor-retinoid X receptor (RAR-RXR), vitamin D receptor-RXR (VDR-RXR) and thyroid receptor-RXR (TR-RXR). Multiple isoforms exist for the protein subunits. RXR proteins, for example, include RXRα, RXRβ, and RXRγ, each associated with different binding patterns and functions [1,3]. RAR proteins include RARα, RARβ, and RARγ. Ligands for the nuclear receptors, both natural and synthetic, are numerous. Examples include vitamin A metabolites (for RAR, RXR, and the peroxisome proliferator-activated receptor β/δ (PPAR β/δ) [4]), 17-β estradiol (for ER), progesterone (for PR), testosterone and dihydrotestosterone (for AR), 3,3′,5,5′ triiodo-L-thyroxine (for the thyroid receptor [TR]), and dexamethasone and prednisolone (for GR). The natural ligand for RXR remains a topic of debate. Although 9-cis retinoic acid has been discovered to bind RXR, this metabolite is difficult to detect in many mammalian tissues. Other possible natural ligands for RXR include unsaturated fatty acids such as docosahexaenoic acid, arachidonic acid and oleic acid [3].

The influences of nuclear receptors and their ligands on the mammalian cell are extraordinarily complex. Vitamin A, for example, signals the mammalian cell as soon as it reaches the plasma membrane [5]. Signals continue as vitamin traffics through the cell toward the nucleus, engaging numerous chaperones along the way. Receptors such as RXR also exhibit cytoplasmic functions, distinct from their activities as transcription factors. Within the nucleus, when vitamin A serves as a ligand for its nuclear receptors, it can instruct conformational changes [1,6,7,8,9]. Ligand-bound and unbound receptors, when associated with DNA (either by direct DNA–protein interactions or by DNA tethering via protein–protein interactions), can recruit, inhibit, and interact with other transcription factors to instruct DNA configurations and gene expression patterns [1].

Class II heterodimers have been further characterized as ‘permissive’ or ‘non-permissive’. The term ‘permissive’ was attributed to heterodimers that were activated in a controlled setting by ligand binding either to RXR, its partner protein (e.g., PPAR), or both. The term ‘non-permissive’ was attributed to heterodimers that could not be activated by ligand binding to RXR alone. Permissive receptor proteins include PPAR, liver X receptor (LXR), farnesoid X receptor (FXR), pregnane X receptor (PXR) and constitutive androstane receptor (CAR), each of which is responsive to diet-derived lipids. Non-permissive receptor proteins include RAR, VDR, and TR [1,10,11,12].

A survey of nuclear receptor DNA-binding patterns has revealed consensus nucleotide motifs (response elements) to which each receptor preferentially binds. As examples, type II receptors often bind two tandem, hexad half-sites, RG(G/T)TCA, separated by a short spacer [13]. RAR-RXR binding to DNA shows a preference for spacers of size 5 (direct repeat 5, DR5) or 2 (DR2), whereas VDR-RXR binding to DNA shows a preference for spacers of size 3 (DR3) and TR-RXR shows a preference for spacers of size 4 (DR4) [1]. Occasionally, the receptors bind individual half-sites or half-sites separated by unusually long spacers. RXR can also function as a self-sufficient homodimer, able to bind a DR1 element. The estrogen receptor often binds a palindromic motif GGTCAnnnTGACC [14,15]. These binding patterns are far from absolute and binding is often observed at sites that lack a canonical sequence [1,7,9,16,17,18,19,20,21,22,23,24,25,26,27,28,29]. Nuclear receptor cross-regulation is supported by receptor sharing of (i) protein partners (RXR is shared among the type II receptors), (ii) ligands (e.g., retinol binds RAR-RXR and PPAR-RXR), and (iii) DNA binding sites.

The outcome of nuclear receptor binding to DNA is difficult to predict due to the high complexity of protein–DNA complexes. For example, the estrogen regulation of the *GREB1* gene involves ERα and RNA polymerase II (RNA Pol II) binding to three different estrogen response elements (ERE) within a 20 kb region and DNA looping that associates EREs with the gene’s transcriptional start site [30,31].

## 2. Antibody Expression by the Mature B Cell

In the developing fetus, the site of mammalian B cell development is the yolk sac. Post-birth, conventional B cells develop in the bone marrow, dependent on bone marrow stroma. Stem cells progress through multiple stages of B cell development. At the pro B cell stage, gene rearrangements are initiated in the immunoglobulin heavy chain locus. Each B cell undergoes unique D-J and V-DJ gene rearrangements with the excision of intervening sequences to create a V-D-J coding sequence. At the pre-B cell stage, the transcription of V-D-J-C mRNA sequences and RNA splicing yield a μ heavy chain protein that can be detected in the cytoplasm or in combination with a surrogate light chain on the B cell surface. In Pre-B cells, there is also a rearrangement of V and J genes within the immunoglobulin light chain loci (к or λ). Once the V-J-C light chains are expressed, two identical heavy chains and two identical light chains join to produce the classical immunoglobulin M (IgM) molecule, now advancing the cell to the immature B cell stage. After these antigen-independent processes occur in the bone marrow, the cells move to the periphery and develop into mature B cells (also called naïve B cells) expressing IgM and immunoglobulin D (IgD) via alternate RNA splicing [32]. B cells are activated when antigen or mitogen engages their cell surface antibodies, at which time B cells proliferate and can mature to antibody-secreting plasma cells and/or memory cells. While B cells are best known for antibody production, they can also regulate other cells of the immune system [33].

After activation, the B cells may undergo somatic mutation and may also switch to immunoglobulin isotypes G, E, and A (IgG, IgE or IgA) by class switch recombination (CSR). The CSR process loops DNA in the immunoglobulin heavy chain locus, cuts DNA at the switch sites, deletes Cμ, Cδ and other intervening sequences, and re-ligates DNA to reposition V-D-J near the Cγ, Cε, or Cα genes. The mechanism of CSR begins with the production of sterile transcripts by RNA Pol II, initiated upstream of the targeted switch sites. Polymerase stalls in the switch regions and recruits activation-induced cytidine deaminase (AID). AID converts cytidine to uracil, followed by the uracil DNA glycosylase (UNG)-mediated removal of uracil. Then, the DNA is cleaved by apurinic/apyrimidinic endonucleases, and non-homologous end joining completes the process [34].

Regulatory regions have been defined that influence immunoglobulin expression and CSR. In mice, the 3′ regulatory region (3′RR) includes multiple DNase I hypersensitive sites (hs3a, hs1,2, hs3b, and hs4) with enhancer activity, situated downstream of Cα. The 3′RR interacts in loop formation with Eμ (a promoter/enhancer upstream of Cμ), and switch regions. When the 3′RR is absent, the mice express low levels of IgM and are deficient in CSR [35,36,37,38]. The hs1,2 sequence is of particular interest, because polymorphisms in this region in humans associate with an increased risk of *systemic lupus erythematosus* (lupus), a disease with a 9:1 female:male ratio [39].

## 3. Sex and the Immune Response

The immune responses of males and females differ [40,41,42,43,44,45,46]. Females generally express more total serum antibodies than males. Females also respond better to influenza virus vaccines and infections compared to males, both in mice and humans [47,48,49]. In humans, estrogen levels have been correlated with IgG responses toward an influenza virus vaccine [50]. Today, there is an unprecedented pandemic of SARS-CoV-2 infections and consequent COVID-19 disease. The influences of sex on this disease are already evident in that males suffer significantly more than females [51,52].

The heightened immune response in females compared to males is not always evident and does not always provide benefit. In fact, males exhibit better responses toward pneumococcus antigens compared to females [48]. Moreover, females often suffer higher frequencies of auto-immune disease compared to males. As noted above, there is a significant female predominance of lupus [53]. When females are pregnant and estrogen levels are extremely high, auto-immune diseases like lupus can be life-threatening [54,55]. Human females additionally suffer from asthma more than males, coincident with increased estrogen levels at the time of female puberty [56].

Using a mouse model for lupus (females of the strain NZM2410), Sven et al. described protection against autoimmune disease upon the knock-out of normal *ERα* expression (although conflicting results have been described [57,58]). In small animal and tissue culture settings, supplemental estrogen increased the antibody levels, including antibodies against self-antigens such as cardiolipin [48,56,59,60,61,62,63,64]. Whereas estrogen enhanced the immunoglobulin expression by human peripheral blood mononuclear cells (PBMC), immunoglobulin production was reduced in the presence of testosterone [64,65]. In a mouse model of allergen-induced dermatitis, an estrogen inhibitor reduced allergen-specific IgG1, IgG2a and IgE, as well as clinical disease symptoms [66].

## 4. Vitamin D and the Immune Response

Vitamin D is ingested from plant (D2) or animal (D3) sources including dairy products (often vitamin fortified) and fish. Vitamin D is also synthesized when 7-dehydrocholesterol in the skin is converted to cholecalciferol upon exposure to sunlight (ultra violet B (UVB)) rays) [67]. In the liver, 25-hydroxylase converts cholecalciferol to 25 hydroxy-vitamin D (25(OH)D, also termed calcidiol), a common metabolite in the blood. Escorts for 25(OH)D in the blood include the vitamin D binding protein (VDBP) and albumin. Polymorphisms in VDBP are common and may influence the efficiency of vitamin D uptake into tissues. Within tissues, 1α hydroxylase converts 25(OH)D to the end-metabolite 1,25 dihydroxy vitamin D (1,25(OH)_2_D, also termed calcitriol). The Office of Dietary Supplements (ODS) in the United States recommends a vitamin D daily intake (recommended dietary allowance, RDA) of 10 μg (~400 international units (IU)) for infants, 15 μg (~600 IU) for individuals of ages 1–70 years, and 20 μg (~800 IU) for individuals of ages >70 years (ods.od.nih.gov). Debates continue as to the appropriate levels of vitamin D in blood, but most scientists agree that blood levels of >30 ng/mL vitamin D will support a healthy immune system.

Vitamin D deficiencies and insufficiencies are recognized throughout the world and affect numerous mammalian systems and conditions including lung development and pregnancy [68]. Nutritional deficiencies occur in both developed and developing countries. In the developed world, vitamin deficiencies are exacerbated by reduced sun exposures and emerging food deserts in low income communities [69].

Vitamin D provides significant benefit to the immune response, both adaptive and innate. For example, when macrophages are infected with *M tuberculosis*, they produce high levels of 1,25(OH)_2_D and effectively kill the pathogen. Vitamin D can also play an important regulatory role by dampening allergic reactions such as asthma, eczema and food allergies or autoimmune disorders such as multiple sclerosis or diabetes mellitus [70,71]. In humans, serum levels of 25(OH)D correlate with total serum IgM and IgG3 [72] and correlate with an improved control of respiratory diseases including tuberculosis [73,74,75].

## 5. Vitamin A and the Immune Response

Vitamin A can be ingested in the form of provitamin A carotenoids from plant foods including green, orange and yellow vegetables such as carrots and sweet potatoes or retinoids (preformed vitamin A) from foods including dairy products, fish, poultry, and other meats (particularly liver). As for vitamin D, the RDA for vitamin A is a topic of continued debate. The ODS recommends a daily intake of vitamin A in retinol activity equivalents (RAE) as approximately 400–500 μg for infants, 300 μg for individuals of ages 1–3 years, 400 μg for individuals of ages 4–8 years, 600 μg for individuals of ages 9–13 years, and 700–1300 μg for individuals of ages >14 years depending on sex, pregnancy, and breastfeeding (0.3 μg RAE = 1 international unit (IU) retinol, ods.od.nih.gov). Blood levels of retinol defined as ‘deficient’ or ‘insufficient’ are also debated. Often, vitamin A deficiency (VAD) is defined as <0.7 μM (~20 μg/deciliter (dL)) retinol and vitamin A insufficiency is defined as ≥0.7, but <1.05 μM retinol. Vitamin A is usually stored in the liver in the form of retinyl esters and traffics through the blood as retinol, chaperoned by retinol binding protein (RBP) in a 1:1 molar ratio. RBP is also bound to a serum protein transthyretin (TTR) which assists retinol delivery to peripheral tissues. Other chaperones for vitamin A in blood or lymph include albumin and chylomicrons. D’Ambrosio et al. have estimated that 25–33% of all retinoids absorbed by the intestine are delivered by chylomicrons or chylomicron remnants to tissues other than the liver (white adipose tissue, skeletal muscle, heart, lungs, and kidneys), explaining the good health of humans and mice that lack RBP [76].

In tissues, retinol can be metabolized to retinal (catalyzed by alcohol dehydrogenase) and then to retinoic acid (RA, catalyzed by retinaldehyde dehydrogenase, ALDH1A (also termed RALDH)), an end-stage metabolite. While alcohol dehydrogenase is produced ubiquitously, ALDH1A is selectively expressed, prominently found among dendritic cells in the gastrointestinal tract and among epithelial cells surrounding the airways [77].

Vitamin A deficiencies (VAD) are common in the developed and developing world, although conditions may go unnoticed and underreported in some countries [78]. In Memphis, TN, USA, a significant fraction of children and adults are VAD or vitamin A insufficient [79].

Similar to other ligands for nuclear receptors, vitamin A exhibits a plethora of functions. Virtually every organ system depends upon vitamin A, and this vitamin has a prominent role in immune protection against infectious diseases. The integrity of epithelial cells that line airways, necessary as a first line of defense against respiratory pathogens, is supported by vitamin A.

In vitamin A-deficient (VAD) mice, we observed poor local IgA responses toward respiratory virus vaccines and poor T cell responses. [80,81]. In VAD mice, both T cells and dendritic cells expressed unusually high levels of CD103 (the αE component of αEβ7, the receptor for e-cadherin), likely contributing to aberrant immune cell trafficking [82,83]. The immune response was further weakened when the mice were rendered double-deficient for vitamins A and D [84]. VAD is also associated with reduced protection against bacterial infections [85,86,87,88] and in a murine B cell acute lymphoblastoid leukemia model (B-ALL), VAD mice exhibited poor tumor clearance compared to vitamin-replete controls [89]. In the context of diet-induced obese (DIO) mice, vitamin A levels were normal or above-normal in the blood, but were low in tissues. DIO mice accordingly exhibited poor immune responses toward an influenza virus vaccine and poor control of a subsequent challenge with influenza virus [90].

Vitamin A was correlated with IgG4 and IgA among Memphians and low levels of vitamin A were associated with poor outcomes among children hospitalized with respiratory syncytial virus (RSV) or human metapneumovirus (hMPV) infections [72,91].

## 6. Thyroid Hormones and the Immune Response

The thyroid hormones 3,3′,5,5′ tetraiodo-L-thyroxine (T4) and 3,3′,5,5′ triiodo-L-thyroxine (T3), like sex hormones and vitamins, may affect multiple mammalian systems and processes including pregnancy and hypersensitivity [92,93]. With regard to the immune response, both innate and adaptive effectors are influenced by thyroid hormones. Cellular concentrations of T3 and T4 are regulated by 1,2 and 3 iodothyronine deiodinases (D1, D2, and D3). T3 binds TRs including TRα1, TRβ1, TRβ2, and TRβ3 to influence gene expression in the nucleus, but similar to other nuclear receptors and ligands, extranuclear signals are also at play. As an example, T4 binds the integrin αvβ3 to activate kinase activities (5′ adenosine monophosphate-activated protein kinase (AMPK), phosphotidylinositol 3-kinase/Akt (PI3-K/Akt), and mitogen-activated protein kinase (MAPK)) in innate immune cells. T3 supports natural killer (NK) cell activation and the production of interferon γ (IFNγ), and a positive correlation has been demonstrated between serum T3 concentrations and NK cell activity in healthy, elderly humans. T3 can additionally support dendritic cell viability, cell maturation, CCR7 expression, cell migration to lymph nodes, and antigen presentation [94]. The relationships between thyroid hormones and immune responses are circular in that abnormal thyroid hormone production will dysregulate the immune response while a dysregulated immune response can attack the thyroid gland to render hormone production abnormal [95].

## 7. Nuclear Receptor Cross Regulation Influences the Immune Response

**Figure 1:**
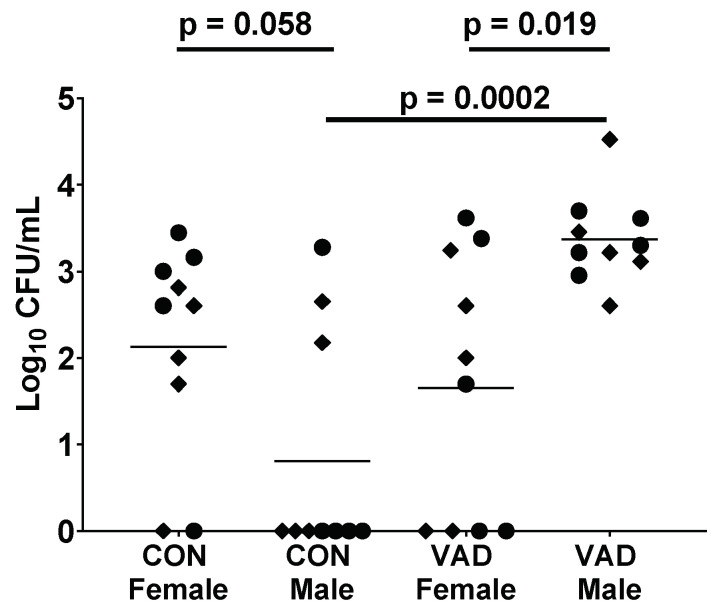
Vitamin A deficient (VAD) male mice exhibit higher bacteria burdens than control males and VAD females. To produce VAD mice, pregnant C57BL/6 (H2-b) mice were purchased from Jackson Laboratories (Bar harbor, ME). Mice were placed on either a control or a VAD diet upon their arrival in the animal facility at St. Jude (days 4–5 gestation). VAD (cat. no. 5WA2, Test Diets) and control (cat. no. 5W9M) diets differed only in vitamin A content, containing either 0 or 15 international units (IU)/g vitamin A palmitate, respectively. Upon reaching adulthood, C57BL/6 control (CON) and VAD mice were lightly anesthetized with isoflurane and intranasally infected with 0.5–1 × 10^5^ CFU of *Streptococcus pneumoniae*, strain A66.1 (as described previously [86,98,99]). Lung bacterial titers were determined 24 h post-infection, assigned the value ‘1′ if below detection. Each dot represents an individual mouse. Results from two experiments are shown, respectively, indicated by circles and diamonds. Significant differences between the paired groups were determined by Mann–Whitney U tests.

Of note, male:female differences in immune patterns are not absolute, but are dependent on genetic and microenvironmental factors including vitamin A. We previously showed that when mice were rendered vitamin A deficient (VAD), isotype profiles changed. For male mice, VAD improved the otherwise low levels of serum IgG2b [48]. In addition, we found that when males were VAD, they lost their advantage over females in terms of protection from a bacterial challenge. This result is shown in Figure 1. Whereas the bacterial burden trended lower in control C57BL/6 males compared to females, the burden was significantly higher in VAD males compared to VAD females. This presumed cross-regulation among nuclear receptors is predicted, given that, as described above, nuclear receptors can share ligands, co-receptors and DNA binding locations. Previous examples of receptor cross-regulation include interactions between ERα and PPAR [96], and ERα and RAR [97].

## 8. Nuclear Receptors, Ligands, and Important Gene Targets among B Cells

It was originally assumed that B cells were only indirect targets of nuclear receptors and their ligands. Direct targets included antigen-presenting cells such as dendritic cells or macrophages [83]. As examples, Mora et al. showed that vitamin A supported the trafficking of dendritic cells to mucosal sites [83] and Hughes et al. showed that progesterone regulated IFNα in dendritic cells [100,101,102,103]. It was then noted that estrogen and progesterone could act directly on B cells by up-regulating AID [102,104,105].

In search of additional, direct influences of nuclear receptors on immunoglobulin expression, we queried the immunoglobulin heavy chain locus for nuclear receptor response elements. We then discovered hotspots for nuclear receptor type I and type II response elements (estrogen response elements (ERE) and retinoic acid response elements (RARE)) in switch sites for Cμ, Cε, and Cα (Sμ, Sε, and Sα) [7,106]. Using CRISPR/Cas9 technologies, we found that the removal of a single estrogen response element (ERE) from Eμ or the 3′RR hs1,2 site in a B cell line reduced the CSR from IgM to IgA [107].

We further observed a partnership of ERα and RNA Pol II [48,107,108,109], a critical component of CSR. When supplemental estrogen was added to purified B cell cultures, sterile transcript levels improved and there were changes in the positions of ERα and RNA Pol II binding within the immunoglobulin heavy chain locus. For both proteins, there was a focus of binding on the ERE hotspot within Sμ. The results suggested that estrogen-liganded ERα served as a chaperone to direct the positioning of RNA Pol II during B cell activation and CSR. These results helped explain, at least in part, the mechanism by which estrogen influenced immunoglobulin expression [109].

**Figure 2:**
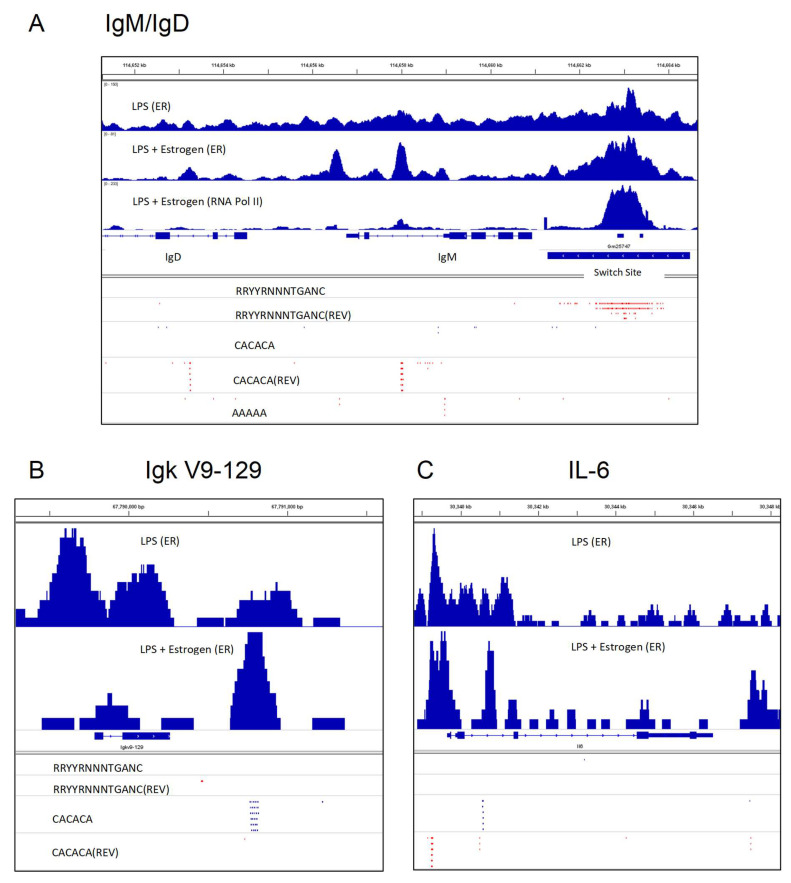
Purified murine B cells were stimulated for 1 day with lipopolysaccharide (LPS) or LPS + supplemental estrogen, followed by chromatin immunoprecipitation studies with antibodies toward ERα (termed ER in this figure) or RNA Pol II, as described previously [48,107,108,109,117]. Briefly, splenic B cells were isolated from adult, C57BL/6 female mouse spleens, by negative selection with anti-CD43 and anti-CD11b antibodies. The cells were then stimulated overnight with LPS with or without supplemental estrogen (100 nM). Immunoprecipitations were with anti-ERα or anti-RNA Pol II antibodies. Integrative Genomics Viewer (IGV) software was used to generate the figures and to identify motifs in forward and reverse (REV) orientations. (**A**) The switch site (Sμ), Cμ and a portion of the Cδ gene fragment are shown from right to left. Potential ER binding sites are shown using the motif RRYYRNNNTGANC (IGV ‘Find motif’ function). The positions of adenosine–cytidine (AC)-repeats (CACACA) and poly A (AAAAA) are shown. (**B**) The Ig kappa V9-129 gene fragment is shown with motifs listed. (**C**) The *IL-6* gene is shown, with the same motifs listed as in (**B**). The detailed methods and results from chromatin immunoprecipitation studies have been described previously [48,106,107,108,109,117].

A snapshot of these ERα and RNA Pol II binding features is illustrated in Figure 2A. This figure was produced using Integrative Genomics Viewer (IGV) software to show ERα (top two rows) and RNA pol II (third row) binding patterns in areas of Sμ, Cμ (IgM) and Cδ (IgD) regions when B cells were stimulated with lipopolysaccharide (LPS) alone (top row) or LPS plus supplementary estrogen (100 nM estrogen, second and third rows). The splenic B cells were purified (by negative selection with antibodies specific for CD43 and CD11b) from C57BL/6 mouse spleens and stimulated in tissue culture overnight before harvest. The red dashes identify the positions of various sequences in forward and reverse (REV) orientations. Figure 2A illustrates the previously described findings that for B cells stimulated in the presence of supplemental estrogen, there was a focused binding of factors on Sμ and also on adenosine–cytidine (AC) repeats in the immunoglobulin heavy chain locus [107,108,109].

Here, we extend our study by focusing on ERα binding shifts in areas near variable gene segments within immunoglobulin heavy- and light-chain loci. In Figure 2B is shown the kappa variable gene segment, Igк V9-129. When supplementary estrogen was added to B cell cultures, ERα binding was more pronounced near the AC repeat downstream of Igк V9-129. Similar patterns were observed for subsets of V_H_, Vλ and Vк genes, in that they were often flanked either upstream and/or downstream by AC repeats and ERα binding was focused on these sites when supplemental estrogen was added to B cell cultures. We consider that shifts in ERα binding patterns might influence non-identical V gene segment transcription rates [110] (Figure 2B).

Shifts in ERα binding to AC repeats in the context of supplemental estrogen were also noted near non-immunoglobulin genes such as *IL-6* (Figure 2C). *IL-6* is known to upregulate following the LPS stimulation of murine B cells, a feature associated with autoimmunity [111,112]. Further experimentation is needed to determine if/how these estrogen-induced shifts of ERα toward the binding of AC-rich regions may alter DNA looping, gene rearrangements, transcription, and/or splicing [110,113,114,115,116] to influence gene expression patterns in developing or activated B cells.

## 9. When B Cells Need Correction

Two extremes of B cell malfunction include (i) an insufficient immune response against pathogens, and (ii) an over-exuberant immune response toward inert antigens or self. In the first case, the host is vulnerable to numerous viral, bacterial, and fungal infections, whereas in the second case, the host suffers from unnecessary immunopathologies. In cases of influenza virus infections and the more recent SARS-CoV-2 infections, both outcomes have been observed, wherein the immune system may clear the virus too slowly, but following virus clearance, immune responses toward damaged tissues and residual viral antigens may exacerbate disease.

Can clinical corrections be made with a focus on nuclear receptors and their ligands? In the case of VAD, vitamin supplementation may provide a simple form of correction and in the case of sex hormones, the use of agonists/antagonists (e.g., estrogen/tamoxifen) may be used. As described above, in VAD mice, we observed poor IgA responses toward respiratory virus vaccines. These could be corrected if vitamin supplements were administered at the time of vaccination [80,81]. In the mouse model of diet-induced obesity, vitamin A supplements supported antibody production and assisted clearance of virus at a later stage when vaccinated obese mice were challenged [90]. The reductions observed among the virus-specific immune responses in mice that were double deficient for vitamins A and D could also be corrected [84]. Again, we found that vitamin supplements, in this case with vitamins A and D, could be administered at the time of vaccination to correct the antibody response toward vaccines [84].

However, despite the apparent simplicity of supplementation methods, caution is advised in the clinical arena. Outcomes of clinical supplementation studies have been highly variable, dependent on the geographical location, diet, sex, and age of study participants. For example, studies have often shown that vitamin A supplements provide benefit in the developing world, but different outcomes for males and females have been noted [118,119,120] with males sometimes exhibiting greater benefit from supplementation compared to females.

The timing of supplementation relative to vaccination or infection also affects outcome. Hussey et al. described a randomized, double-blind clinical study in which children hospitalized with measles were administered either a total oral dose of 400,000 international units (IU) retinyl palmitate or a placebo control [121]. The authors found that the children who received the vitamin recovered more rapidly from pneumonia and diarrhea compared to the controls and experienced shorter hospital stays [120,121,122]. In contrast, Bresee et al. demonstrated that vitamin A supplements worsened the outcome when given to children hospitalized with respiratory syncytial virus (RSV) infections in the United States [123]. Our own study of influenza virus vaccinations in 2–8 year old children in Memphis, TN, showed that baseline vitamin A levels correlated positively with the vaccine-induced immune response whereas baseline vitamin D levels correlated negatively. When vitamin A + D supplements were administered to these children at the time of vaccination, there were improved responses compared to placebo controls, but only when children had low vitamin A and D levels at baseline. The responses were worsened by vitamin A + D supplementation when the children were vitamin replete at baseline [79]. In a separate study in Indonesia, six month-old infants received a vitamin A supplement at the time of measles vaccination; at twelve months the supplemented children had a lower sero-conversion rate toward the vaccine compared to the controls [124,125,126]. This result contrasted with that of a study of nine month-old children given vitamin A supplements at the time of a single measles vaccine dose in Guinea Bissau. Then, the geometric mean titers toward the vaccine were significantly improved in the supplemented compared to the control children at 18 months, particularly for boys [125,127].

Like vitamin supplements, thyroid and sex hormone supplements have yielded variable results in the clinic. When T3 was administered to elderly participants to increase NK activity, improvements were only indicated when baseline T3 concentrations were low [128]. The selective estrogen receptor modulator (SERM) tamoxifen exhibited both supportive and inhibitory functions, depending on the tissue target [129] and the benefit of estrogen replacement therapy in post-menopausal women to improve vaccine and virus-specific immune responses has been a topic of continued debate [130,131,132,133,134,135].

The results described above demonstrate that supplementation cannot be implemented globally using a one-size-fits-all strategy. Rather, the fine-tuning of supplementation programs based on population genetics and microenvironments may be required to yield predictable and beneficial outcomes.

Today, new technologies offer new treatment options. There are now a variety of methods for blocking protein interactions with DNA or RNA (e.g., CRISPR-CAS9 technologies, anti-sense oligonucleotides, DNA decoys or small inhibitory molecules [113,116,136,137,138,139,140,141,142,143,144,145,146,147,148,149]). Perhaps fine-tuned and targeted manipulations of nuclear receptor binding sites within promoters, enhancers and switch sites of the immunoglobulin loci will ultimately prove successful for the control and optimization of immunoglobulin expression.

## Abbreviations

1,25 (OH)2D	1,25-dihydroxy vitamin D
25(OH)D	25-hydroxy vitamin D
VAD	Vitamin A deficient
ER	Estrogen receptor
ChIP	Chromatin immunoprecipitation
DIO	Diet induced obesity
RAR	Retinoic acid receptor
PPAR	Peroxisome proliferator activated receptor
RXR	Retinoid X receptor
